# A Decreased Frequency of Regulatory T Cells in Patients with Common Variable Immunodeficiency

**DOI:** 10.1371/journal.pone.0006269

**Published:** 2009-07-29

**Authors:** Karina M. Melo, Karina I. Carvalho, Fernanda R. Bruno, Lishomwa C. Ndhlovu, Wassim M. Ballan, Douglas F. Nixon, Esper G. Kallas, Beatriz T. Costa-Carvalho

**Affiliations:** 1 Federal University of São Paulo, São Paulo, Brazil; 2 Division of Experimental Medicine, San Francisco General Hospital, University of California San Francisco, San Francisco, California, United States of America; 3 University of São Paulo, São Paulo, Brazil; New York University School of Medicine, United States of America

## Abstract

**Introduction:**

Common variable immunodeficiency disorder (CVID) is a heterogeneous syndrome, characterized by deficient antibody production and recurrent bacterial infections in addition abnormalities in T cells. CD4^+^CD25^high^ regulatory T cells (Treg) are essential modulators of immune responses, including down-modulation of immune response to pathogens, allergens, cancer cells and self-antigens.

**Objective:**

In this study we set out to investigate the frequency of Treg cells in CVID patients and correlate with their immune activation status.

**Materials and Methods:**

Sixteen patients (6 males and 10 females) with CVID who had been treated with regular intravenous immunoglobulin and 14 controls were enrolled. Quantitative analyses of peripheral blood mononuclear cells (PBMC) were performed by multiparametric flow cytometry using the following cell markers: CD38, HLA-DR, CCR5 (immune activation); CD4, CD25, FOXP3, CD127, and OX40 (Treg cells); Ki-67 and IFN-γ (intracellular cytokine).

**Results:**

A significantly lower proportion of CD4^+^CD25^high^FOXP3 T cells was observed in CVID patients compared with healthy controls (*P*<0.05). In addition to a higher proportion of CD8^+^ T cells from CVID patients expressing the activation markers, CD38^+^ and HLA-DR^+^ (*P*<0.05), we observed no significant correlation between Tregs and immune activation.

**Conclusion:**

Our results demonstrate that a reduction in Treg cells could have impaired immune function in CVID patients.

## Introduction

Common variable immunodeficiency disorder (CVID) is the most frequent symptomatic primary immunodeficiency, characterized by recurrent bacterial infections, hipogamaglobulinemia and impaired antibody responses [1]–[4]. CVID patients usually present recurrent respiratory infections, and elevated incidence of autoimmune, gastrointestinal, lymphoproliferative, and granulomatous diseases [5]–[7]. Although genetic defects associated with CVID have been described, they are rare [7]–[9].

Over the last 20 years various facets of cellular and immunological dysfunctions in CVID have been described, for example, abnormalities in B cell populations, low frequencies of naive CD4 T cells, and an increase in cellular activation [10]–[14]. A new classification of this disease has recently been proposed according to the B cell phenotype [15]–[17]. However, a significant number of studies have shown alterations in phenotype and function of T cell subpopulations [13], [18], [19], [20].

T cell functional defects compromise T cell activation and proliferation [12], [18]. Abnormalities in the secretion of cytokines [13], [21]–[23], defects in early T cell receptor (TCR) signaling events [10], [24], and impaired expression of activation markers including CD40-ligand, attractin and L-selectin [25]–[27] have been described. In addition, current studies have described a breakdown in mechanism of tolerance in CVID subjects with autoimmunity [28].

Tolerance to self antigens is an active process that has central and peripheral components [29]. Among T cell subsets, a subgroup of CD4^+^ T cell, referred to as regulatory T cells (Tregs), has an important role in controlling other immune responses, maintaining peripheral self-tolerance [29], [30]. Treg cells are characterized by the expression of CD25 and the Forkhead family transcription factor (FOXP3) [29], [31] and play a significant role in down regulating the immune response to a variety of non-self antigens, such as microbial, tumor and alloantigens [31]–[33].

Tregs are dysfunctional in several human diseases [30], [34] and are a potential target for therapeutic modulation [30], [35]. In CVID, recent reports describe a decreased frequency of Treg cells [28], [36]. Here, we investigate the frequency of Tregs in CVID patients, and their relationship to cell activation status.

## Materials and Methods

### Subjects

Sixteen patients with a diagnosis of CVID according to the criteria established by the Pan-American Group for Immunodeficiency (PAGID) [37] and, fourteen healthy controls were enrolled in the study. The patients were recruited at the Division of Clinical Immunology at UNIFESP (Sao Paulo, Brazil). All patients were on regular intravenous immunoglobulin (IVIG) substitution therapy. For each subject, complete blood counts and lymphocyte subsets were performed, and the blood sample was collected immediately before IVIG infusion. Clinical information was collected by questionnaire based on the medical charts. The subjects' characteristics are shown in Table 1. Among patients with autoimmunity two were receiving oral corticosteroids, in a dosage less than 0.5 mg/Kg on alternative days. After approval by the local institutional review board (IRB, Comitê de Ética em Pesquisa da Universidade Federal de São Paulo/UNIFESP), written informed consent was obtained from all participants or their legal representatives.

**Table 1:** Clinical and laboratory characteristics of studied subjects.

	Controls	Patients	*P* -value
	(n = 14)	(n = 16)	
Age in years (median, IQR)	28 (13–30)	24 (16.5–31.2)	
Gender (female %)	64%	63%	
Age at diagnosis in years (median, IQR)	_	22 (13–26)	
Age at first symptoms in years (median, IQR)	_	12 (3–16)	
Delayed to diagnosis in years (median, IQR)	_	9 (4–12)	
Autoimmune complications (n, %)	_	6 (37.5%)	
Chronic pulmonar disease (n, %)	_	12 (75%)	
IgG mg/dl (median, IQR)	_	574 (481–1052)	
WBC (cells/mm^3^, median, IQR)			
Leucocytes	5,690 (5,295–7062)	7,185 (6,032–9432)	0.047
Neutrophils	2,976 (2140–3529)	4,490 (3,793–5484)	0.01
Monocytes	463 (409–505)	531 (314–627)	0.69
Lymphocytes	2,296 (1,878–2,493)	1,638 (1,204–2,128)	0.59
T CD3^+^	1,735 (1,367–1,838)	1,226 (1,021–1,842)	0.83
T CD4^+^	918 (707–1000)	550 (477–875)	0.16
T CD8^+^	556 (504–628)	803 (583–414)	0.19
CD4^+^: CD8^+^ T cell ratio	1.64 (1.37–1.8)	0.95 (0.77–1.36)	0.01
B cell phenotyping according to EUROCLASS			
smB+21normal		6 (37.5%)	
Smb+21low		7 (43.7%)	
smB-21low		2 (12.5%)	
B-		1 (6.2%)	

IQR: Inter -quartile range.

### Sample collection

Peripheral blood mononuclear cells (PBMC) were isolated by density-gradient sedimentation using Ficoll-Paque (Pharmacia Biothec, Upssala, Sweden). Isolated PBMC were then washed twice in Hank's balanced salt solution (Gibco, Grand Island, NY). Cells were cryopreserved in RPMI 1640 (Gibco), supplemented with 20% heat-inactivated fetal bovine serum (FBS; Hyclone Laboratories, Logan UT), 50 U/ml of penicillin (Gibco), 50 µg/ml of streptomycin (Gibco), 10 mM glutamine (Gibco) and 7.5% dimethylsulphoxide (DMSO; sigma, St Louis, MO). Cryopreserved cells from all subjects (patients and controls) were stored in liquid nitrogen for a mean time of 2.5 months (1–5 months) until used in the assays. On the day of assay, PBMC were rapidly thawed in a 37°C water bath and washed in RPMI 1640 supplemented with 10% fetal calf serum, 100 U/ml of penicillin, 100 µg/ml of streptomycin and 20 mM glutamine (R10). Cells were counted, checked for viability and resuspended in R10 at concentration of 10 ^6^cells/ml.

### Flow cytometry

The following monoclonal antibodies were used in the assays: CD3- peridin chlorophyll protein (PerCP) and CD4- allophycocyanincarbocyanin 7 (APC-Cy7), and in addition to: HLA-DR- FITC, CD38- PE, CD8-APC, CCR5- PeCy7 (panel A); OX40-fluorescein isothiocyanate (FITC), CD127- phytoerythrin (PE), FOXP3- allophycocyan (APC), CD25- phycoerithrincarbocyanin (PE-Cy7) (panel B); intracellular staining for cytokines was performed using mouse anti-human Ki-67- FITC mouse anti-human interferon (IFN -γ) -PE-Cy7 Ki-67-FITC, all from BD PharMingen (panel C). Fluorescence-minus-one (FMO) was used for gating strategy [38].

Thawed PBMC were centrifuged at 1500 g for 15 minutes and transferred into V-bottom 96-well plates (Nunc, Roskilde, DenmarK) in 200 µl of staining buffer [phosphate-buffered saline (PBS) supplemented with 0.1% sodium azide (Sigma) and FBS, pH 7.4–7.6)] with the panel of surface monoclonal antibodies. Cells were incubated at 4°C in darkness for 30 minutes, washed twice and then resuspended in 200 µl of fixation buffer [1% paraformaldehyde (Polyscience, Warrigton, PA) in PBS, pH 7.4–7.6.

For panel B, cells were resuspended in 50 µl of staining buffer with monoclonal antibody FOXP3, then were incubated at 4°C in darkness for 30 min, washed twice and resuspended in 200 µl of 2% of paraformaldehyde (PFA). For panel C, intracellular staining was performed after surface with CD3- PerCP, CD4-APC-Cy7 and CD8-APC. Cells were incubated with 100 µl of 4% fixation buffer and washed with permeabilization buffer (PBS supplemented with 0.1% sodium azide, 1% FBS and 0.1% saponin; Sigma). Each sample was resuspended in 100 µl of permeabilization buffer, incubated for 15 minutes at room temperature in darkness, washed with 100 µl of staining buffer and incubated for 30 minutes at 4°C in darkness with either without antibody (unstained tube) or anti-Ki67–FITC, anti- IFN-γ–PE–CY7 in 50 µl of staining buffer. Cells were washed with 170 µl of staining buffer and resuspended in 100 µl of 1% paraformaldehyde (PFA) for flow cytometry analysis. Samples were acquired on a FACSCanto, using FACSDiva software (BD Biosciences), and analyzed with FlowJo software (Tree Star, San Carlo, CA). Fluorescence voltages were determined using matched unstained cells. Compensation was carried out using CompBeads (BD Biosciences) single stained with CD3-PerCP, CD4-APC-CY7, CD4-PE-CY7, CD3-PE or CD3-APC. Samples were run for a minimum of 300,000 events in a live lymphocyte gate.

### Statistical analyses

Groups were compared using non-parametric models; data are reported as median and interquartile range (IQR). Comparisons among groups were carried out using the Mann Whitney test. Correlations were performed using the Spearman non-parametric test. *P* - values were considered significant if below 0.05.

## Results

### CD8+ T lymphocytes are highly activated in patients with CVID

**Figure 1:**
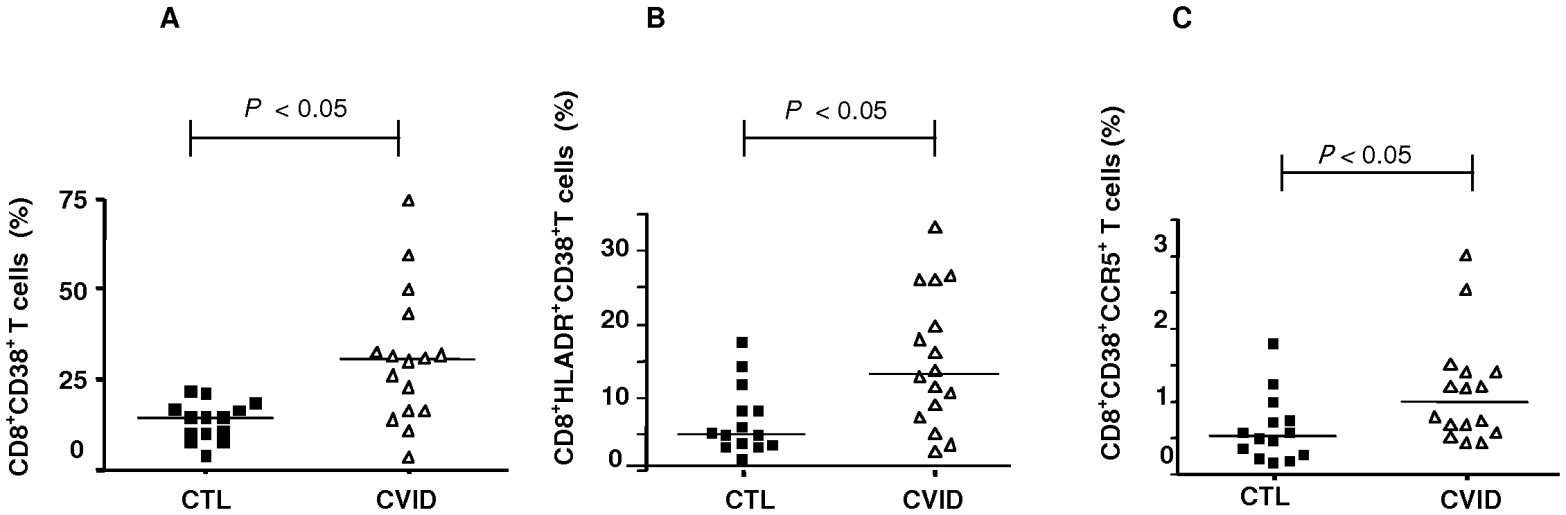
Comparisons of CD8^+^CD38^+^ (A), CD8^+^HLA-DR^+^ (B) and CD8^+^CD38^+^CCR5^+^ (C) T cell percentages in PBMC in healthy controls (CTL) and CVID patients showing significant difference in the activation. Figure 1A-, *P* = 0.002; Figure 1B-, *P* = 0.008; Figure 1C, *P* = 0.021.

We assessed the characteristics of the lymphocyte subsets in 30 subjects, among CVID patients and healthy controls. Further analysis using a combination of activation markers showed a marked up-regulation of CD38^+^ and CCR5^+^ expressing CD4^+^ T cells in the CVID patients (*P* = 0.04) (data not shown) and an increased frequency of CD38^+^ expressing CD8 T cells in CVID patients compared to controls (*P* = 0.002). Both high co-expression levels of HLA-DR^+^ (*P* = 0.008) or CCR5^+^ (*P* = 0.021) confirmed the high level of CD8 T cell activation in CVID patients, (Figure 1A–C). Four out of 16 patients had a marked increase in T cell activation. Of these, just one, 24 year old male, presented autoimmune hepatitis, the others had no particular associated pathology.

### Circulating Tregs are diminished in patients with CVID

**Figure 2:**
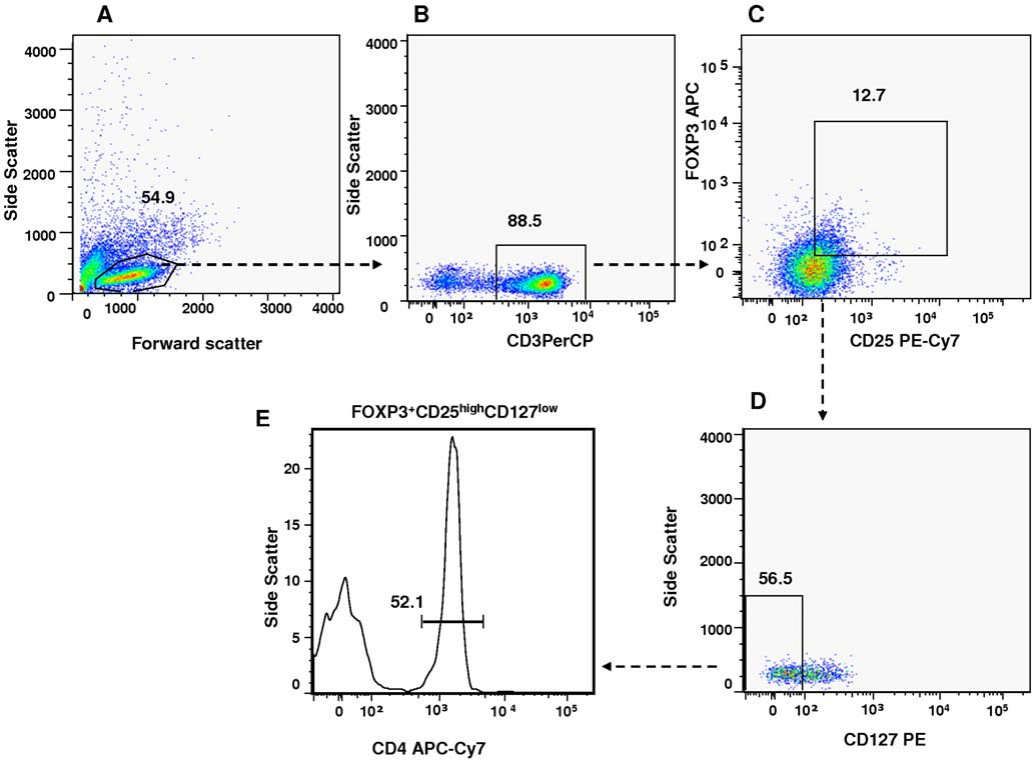
Flow cytometric panels showing the gating strategy from healthy and CVID patients. (A) The gate is set around the population of lymphocytes; (B) CD3 T cells that were evaluated for (C), FOXP3, CD25; (D) Side scatter and CD3^+^FOXP3^+^CD25^high^CD127^low^. (E) The gate is set around the population of FOXP3+CD25^high^CD127^low^CD4^+^ T cells. Fluorescence minus one was used to define the gate used. Arrows indicate the gated population subsequently analyzed.

**Figure 3:**
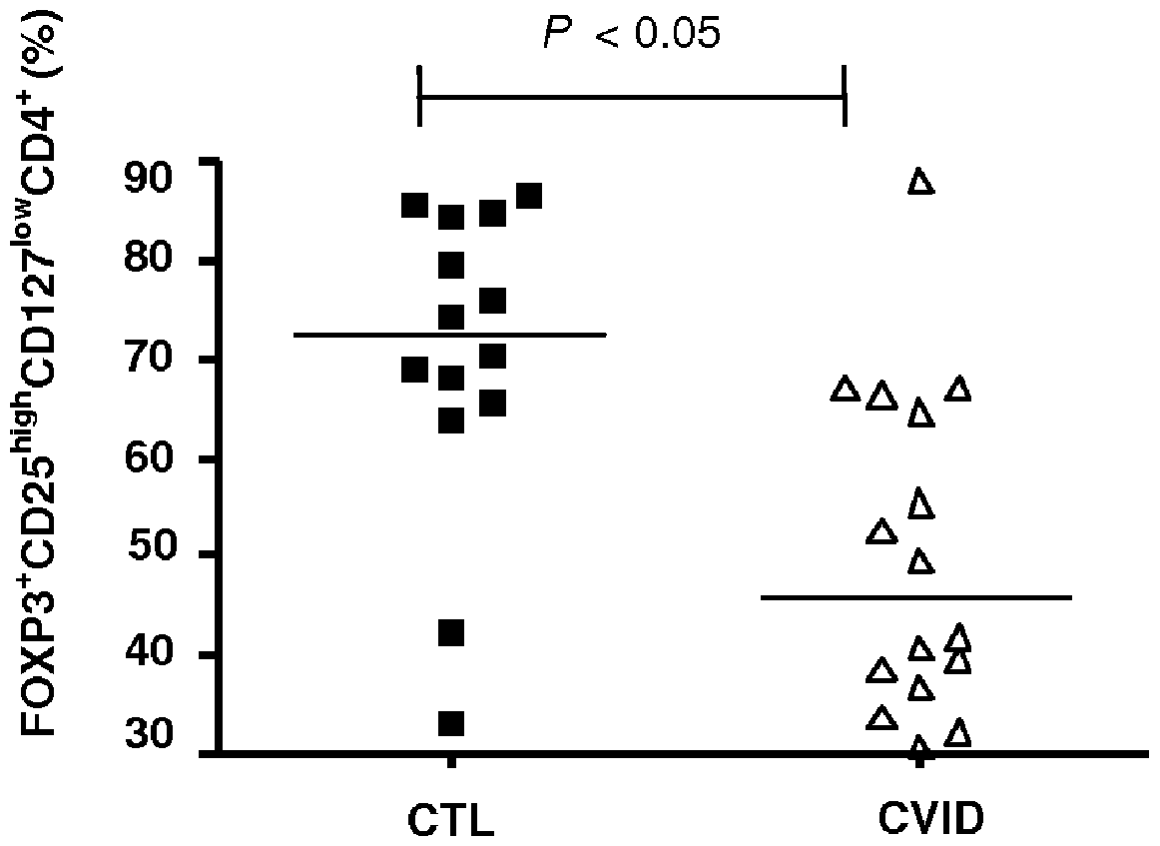
Comparison of Treg cell subsets between healthy controls (CTL) and CVID patients. In CVID group there was a significant lower percentage of FOXP3^+^CD25^high^CD127^low^CD4^+^ T cell (*P* = 0.0034).

We defined Tregs by a series of gating strategies that best represent this suppressor T cell population (FOXP3^+^CD25^high^CD127^low^CD4^+^ T cells) Figure 2. In Figure 3, we show that the frequency of Tregs was markedly reduced in CVID patients, (*P* = 0.03 and *P* = 0.003) compared to healthy controls (Figure 2).

Interestingly, stratifying our patients based on autoimmune status did not reveal any differences in Tregs frequency (data not shown). A similar result was observed when we classified CVID patients according to EUROclass [16] (data not shown). The impact of Tregs and immune activation is well documented [44], [46], however in a regression analysis, we did not observe any association between CD4^+^ or CD8^+^ T cell activation status and the frequency of Tregs in this cohort (data not shown).

### Higher activation was not reflected in higher spontaneous production of IFN-gamma and expression of Ki-67

Presumably due to the high level of T cell activation in CVID, we predicted this would lead to an elevated level of T cell functionality. We thus assessed CD4^+^ and CD8^+^ T cell proliferation by measuring Ki-67 nuclear antigen expressed in the G_1_, S, G_2_, and M phases, but not in the G_0_ phase, of the cell cycle. We also assessed for spontaneous IFN-gamma production in the absence of any stimuli in the CVID cohort. No difference was observed in either Ki-67 expression or spontaneous IFN-gamma production compared to controls subjects (data not shown).

## Discussion

Previous reports have demonstrated abnormalities in cellular immunity on CVID pathogenesis that affect activation and proliferation of T cells and, consequently, B cell differentiation and production of antibody [10], [12], [19], [39]. However, there are few studies examining the influence of Tregs in CVID subjects [36], [40].

We observed that our patients presented significantly higher levels of CD8^+^ T cell activation. The percentage of Tregs expressing CD25^high^FOXP3^+^ was found to be lower in CVID subjects compared to controls but no significant association between Treg cells and immune activation was observed in these patients.

One of the greatest difficulties in studying Tregs is to select the combination of markers that best describe these subsets and currently the most common markers used to define these cells are CD25^high^ and FOXP3^+^ [41]–[43]. The absence of IL-7R (CD127) on CD4 T cells has proven to be a reliable delineator of selection for Treg cells with the highest suppressive function [44]–[46]. Our results using these markers are in line with previous reports, showing a reduced number of Treg cells in CVID patients [28], [36]. It is important to emphasize that these results were observed in a small group of Brazilian patients with different demographic and clinical characteristics to European subjects, which suggests that the proportion of Tregs could be directly related to this immunodeficiency.

Fevang et al. described that CVID patients with splenomegaly had a lower proportion of Tregs compared to others CVID patients [36], as our study, just included one patient with splenomegaly, this suggests that others clinical complications must be associated with reduced Treg population. One topic for further discussion is whether this low percentage is the cause or consequence of clinical complications observed in CVID patients.

No differences were observed in Tregs frequency when comparing CVID patients with and without AID. Despite preliminary studies having suggested an association between these cells and AID in CVID [40], it has not been established and can be influenced by other associated clinical complications that affect not only the frequency, but the profile of cytokine secreted by Tregs.

All patients analyzed were being treated with IVIG and, the correlation between Tregs and IVIG has already been described showed and expansion of Tregs in animal models [47], however we observed a reduced frequency of these cells in CVID subjects. Also, it has been demonstrated that Tregs increases the intracellular expression of TGF-β, IL-10 and FOXP3 following the addition of IVIG [48], but unfortunately we did not analyze the cytokine secretion by Tregs in our patients.

Furthermore, considering the increase of T cell markers activation in CVID subjects, specially CD8+ T cells, without a significant increase in IFN-g production, we suggest that these cells could have a suppressor function, according to previous reports and this result probably was unaffected by IVIG replacement [22], [49]. The similar percentage of cells expressing proliferation marker (Ki67) between the two groups could reflect the absence of lymphoproliferative disease in our sample, since contrasting results have been described in the literature [12], [23].

To summarize, this paper shows that CD25^high^FOXP3^+^CD127^low^ expressing Treg cells were lower in CVID patients, which suggests that Tregs cells can be impaired in CVID, thus having influence on the mechanism pathogenic of this complex immunodeficiency.
